# Rho GDP-Dissociation Inhibitor 2 Inhibits C-X-C Chemokine Receptor Type 4-Mediated Acute Lymphoblastic Leukemia Cell Migration

**DOI:** 10.3389/fonc.2020.01512

**Published:** 2020-08-07

**Authors:** Jixian Luo, Junting Wang, Huiguang Zheng, Lan Wang

**Affiliations:** School of Life Sciences, Shanxi University, Taiyuan, China

**Keywords:** RhoGTPases, migration, leukemia, chemokines, receptor

## Abstract

Although we currently have a good understanding of the role C-X-C chemokine receptor type 4 (CXCR4) plays in T cell acute lymphoblastic leukemia (T-ALL), the mechanism of CXCR4-mediated T-ALL migration remains elusive. Therefore, we focus on the downstream signals of CXCR4 that contribute to T-ALL cell migration in this study. Rho GDP-dissociation inhibitor 2 (RhoGDI2) is expressed preferentially in lymphocytes. It interacts with and regulates the activation of Rho proteins by inhibiting the dissociation of GDP and the binding of GTP. In a previous study, we demonstrated that RhoA and RhoC are activated and required for CXCR4-mediated JURKAT cell migration. In the present work, we investigate the role of RhoGDI2 in CXCR4-mediated T-ALL cell migration. Results show that RhoGDI2 sh2 significantly releases its inhibition effects on T-ALL cell migration toward CXCL12 (C-X-C motif chemokine ligand 12). Phosphorylation of RhoGDI2 on Y24 and Y153 releases RhoA and RhoC from RhoGDI2, which recovers CXCR4-mediated migration toward CXCL12 although the phosphorylation of Y130 has less effect on RhoA or RhoC binding. Furthermore, Src is activated by CXCL12. Transfection of siRNAs to Src reduces CXCR4-mediated migration. Src is required for the phosphorylation of RhoGDI2 on Y153, and ABL1 is activated by CXCL12 and responsible for the phosphorylation of RhoGDI2 on Y24 and Y130. Similarly, knockdown of the expression of ABL1 by siRNAs reduces the CXCR4-mediated migration. Therefore, RhoGDI2 may be a brake for CXCR4-positive T-ALL migration. Because migration is a prerequisite for infiltration of leukemia, this work may suggest the possible involvement of RhoGDI2 in infiltration of T-ALL.

## Introduction

Acute lymphoblastic leukemia (ALL) accounts for 70–80% of all leukemia (1). Although the development of clinical treatments have resulted in a cure rate of about 85–90% (2), relapses occur in about 15–20% of children and are still the major reason for early age mortality (3). CXCR4-mediated infiltration of the central nervous system is a severe trait of T cell acute lymphoblastic leukemia (T-ALL) and correlates with relapses (4). Accumulating data have demonstrated that high expression of CXCR4 is associated with infiltration into spleen, liver, lymph nodes, and CNS in T-ALL (4–8). In the critical process of infiltration, transendothelial migration of leukemic cells is required to exit the blood stream into target organs, which is the prerequisite for infiltration (9). Therefore, the mechanism of CXCR4-mediated migration of T-ALL cells is focused on in the following study.

Rho GDP-dissociation inhibitor 2, RhoGDI2, alternative name Ly-GDI or Rho-GDI beta, is expressed preferentially in lymphocytes (10). It interacts with the Rho proteins, including RhoA (11), Cdc42 or Rac1 (12), and Rac2 (13), and regulates the activation of Rho proteins by inhibiting the dissociation of GDP and the subsequent binding of GTP (10, 14). Therefore, it regulates reorganization of the actin cytoskeleton mediated by Rho family members (15). RhoGDI2 high expression has been found in ALL cell lines, for example, the JURKAT T-ALL cell line (16). However, the function of RhoGDI2 in ALL migration remains largely unknown. In this paper, we design experiments to investigate the role of RhoGDI2 in CXCR4-mediated T-ALL cell migration.

## Materials and Methods

### Cell Lines

JURKAT (Clone E6-1) and CCRF-CEM cell lines were kindly provided by Stem Cell Bank, Chinese Academy of Sciences. HEK293T cells were purchased from the American Type Culture Collection (ATCC).

### Reagents or Antibodies

AMD3100 (S8030, Plerixafor, antagonist against CXCR4) was purchased from Selleckchem. Poly-D-lysine (C0312) was purchased from Beyotime Biotechnology. ATP (disodium salt) (IA0590) was purchased from Solarbio.

Recombinant human Src protein (ab79635, His tag active Src) was purchased from Abcam. Recombinant human/feline/rhesus macaque (350-NS) was purchased from R&D systems.

Antibody to human CXCR4 (ab181020) and anti-GFP antibody (ab6556) were purchased from Abcam. Antibodies to RhoGDI2 (sc-271042) and ABL1 (sc-56887) were purchased from Santa Cruz biotechnology. Anti-RhoGDI2 (D262966) for Western blot assay, anti-ACTB (D110001), anti-Src antibody (D221267), and anti-GST-Tag pAb (AE006) were purchased from Sangon Biotech Company. Rabbit mAb to phospho-Src family (Tyr416) (D49G4), rabbit mAb to RhoA (67B9), and rabbit mAb to RhoC (D40E4) were purchased from Cell Signaling Technology. Rabbit mAb to phospho-Lck family (Tyr394) (D155064) was purchased Sangon Biotech. Mouse anti-His-Tag mAb (AE003) was purchased from ABclonal. Monoclonal antibody to phosphotyrosine produced in mouse (PY20, P4110) was purchased from Sigma-Aldrich. Fast Mutagenesis System (K11209) was purchased from TransGen Biotech.

### rDNA Constructs

To construct GST-fused Crk-CTD domain plasmids, CRK-CTD was amplified by PCR using the cDNA from JURKAT cells as a template and cloned into the vector pGEX-4T-1. The open reading frame of the inserted DNA was confirmed by sequencing, and the expression of the GST-fused proteins was verified by SDS-PAGE. Plasmids expressing wild-type (WT) GST-tagged human RhoGDI2 and mutants (Y24F, Y130F, and Y153F) in pGEX-6p-1 vector, GFP-tagged WT human RhoGDI2 in lentiviral pWPXLd vector, and shRNAs targeting RhoGDI2 or random control in lentiviral pDSL_hpUGIP vector were kindly provided by Professor XL Zeng from Northeast Normal University, Changchun, China. Mutants expressing GST-tagged RhoGDI2 Y24E, Y130E, and Y153E and GFP-tagged RhoGDI2 Y24E, Y130E, and Y153E were amplified by PCR using point mutation kits of the Fast Mutagenesis System (K11209) from TransGen Biotech. In this PCR, WT RhoGDI2 plasmid was the template, and primers are shown in Table 1. Mutants expressing GST-tagged RhoGDI2 Y24E130E, RhoGDI2 Y24E153E, RhoGDI2 Y130E153E, and RhoGDI2 Y24E130E153E were amplified similarly by two or three rounds PCR using RhoGDI2 Y24E or Y130E as templates, and the mutants were verified with sequencing.

**TABLE 1:** siRNA sequences or primers designed to construct the corresponding plasmids.

Name	Sequence
control (for RhoGDI2 sh2)	5′GATCCCCTTCTCCGAACGTGTCACGTTTCAAGAGAACGTGACACGTTCGGAGAATTTTTC3′
	5′TCGAGAAAAATTCTCCGAACGTGTCACGTTCTCTTGAAACGTGACACGTTCGGAGAAGGG3′
RhoGDI2 sh2	5′GATCCCCTAGTTCAGCACACCTACATTCAAGAGATGTAGGTGTGCTGAACGTATTTTTC3′
	5′TCGAGAAAAATACGTTCAGCACACCTACATCTCTTGAATGTAGGTGTGCTGAACGTAGGG3′
control (for CXCR4 shRNAs)	5′TGCCTTCTCCGAACGTGTCACGTTTCAAGAGAACGTGACACGTTCGGAGAATTTTTC3′
	5′TCGAGAAAAATTCTCCGAACGTGTCACGTTCTCTTGAAACGTGACACGTTCGGAGAACA3′
CXCR4 sh1	5′TGGTGGTCTATGTTGGCGTCTGTTCAAGAGACAGACGCCAACATAGACCACCTTTTTTC3′
	5′TCGAGGAAAAAAGGTGGTCTATGTTGGCGTCTGTCTCTTGAACAGACGCCAACATAGACCACCA3′
CXCR4 sh2	5′TGGCAGTCCATGTCATCTACTTCAAGAGAGTAGATGACATGGACTGCCTTTTTTC3′
	5′TCGAGGAAAAAAGGCAGTCCATGTCATCTACTCTCTTGAAGTAGATGACATGGACTGCCA3′
RhoGDI2 (Y24E)	5′TGGACAGCAAGCTCAATGAAAAGCCTCCACCA3′
	5′TTCATTGAGCTTGCTGTCCAGCTCATCA3′
RhoGDI2 (Y130E)	5′TACGTTCAGCACACCGAAAGGACTGGGGT3′
	5′TTCGGTGTGCTGAACGTATTTCAGGCCT3′
RhoGDI2 (Y153E)	5′CCTCGGCCTGAGGAGGAAGAGTTCCTCA3′
	5′TTCCTCCTCAGGCCGAGGTCCATAGCTGCCA3′
siRNA control	5′UUCUUCGAACGUGUCACGUTT3′
	5′ACGUGACACGUUCGGAGAATT3′
Src siRNA1	5′GCGGUUACUGCUCAAUGCATT3′
	5′UGCAUUGAGCAGUAACCGCTT3′
Src siRNA2	5′GCGGUUACUGCUCAAUGCATT3′
	5′UGCAUUGAGCAGUAACCGCTT3′
ABL1 siRNA1	5′GCAUUUGGAGUAUUGCUUUTT3′
	5′AAAGCAAUACUCCAAAUGCTT3′
ABL1 siRNA2	5′GGGUGUACCAUUACAGGAUTT3′
	5′AUCCUGUAAUGGUACACCCTT3′
Lck siRNA1	5′CCGAACCCUGGUUCUUCAATT3′
	5′UUGAAGAACCAGGGUUCGGTT3′
Lck siRNA2	5′GGUCCGCCAUUACACCAAUTT3′
	5′AUUGGUGUAAUGGCGGACCTT3′

For CXCR4 shRNA preparation, annealed double-stranded shRNA oligonucleotides shown in Table 1 were cloned into the *Xho*I and *Hpa*I cloning sites of lentiviral pLL3.7 vector with GFP expressing sequence.

### Generation of Lentiviruses and Transfection

RhoGDI2 WT, Y24E, Y130E, and Y153E in pWPXLd and CXCR4 shRNAs in pLL3.7 or RhoGDI2 shRNA2 in pDSL_hpUGIP were transfected together with psPAX2 and pMD2 (at a ratio of 4:3:1) into HEK293T cells with lipofectamine 2000 (Invitrogen) to generate lentiviruses. Viral stocks were made to infect Jurkat cells or CCRF-CEM cells. After 72 h, cells were collected, and cell lysate was used in the following Western blot assay to detect interfering efficiency.

### Small Interfering RNA

JURKAT or CCRF-CEM cells, 1.5 × 10^6^, were seeded in each well of a 24-well plate precoated with 0.1 mg/ml poly-D-Lysine cultured in 500 μl RPMI 1640 medium without antibiotics in a 37°C incubator containing 5% CO_2_ for 20–24 h. Cells attached to poly-D-Lysine-coated wells were washed three times with PBS and then transiently transfected with small interfering RNAs (siRNAs) targeting *Src, ABL1*, or control siRNA using Lipofectamine 2000 following the manufacturer’s instructions for adherent cells. Four to 6 h after transfection, cells were replaced with complete medium. SiRNAs were used at 200 pM. After 48 h, cells were collected for the following Western blot assay to detect interfering efficiency or transwell assay.

### Transwell Assay (*in vitro* Migration Assay)

The migration of JURKAT or CCRF-CEM cells was evaluated using a transwell chamber with 3-μm pores (Corning). Cells were starved in medium containing 0.5% FBS overnight. Then 3 × 10^5^ cells were seeded in the upper wells, and 25 ng/ml CXCL12 was placed in the lower wells. The plates were incubated at 37°C for 4 h with 5% CO_2_. After each experiment, each upper well was gently taken away, and eight pictures of the cells that had migrated to the lower wells were taken randomly under a fourfold objective of a phase contrast microscope. Relative migration rate was the result of dividing the cell number of each group by that of the control group.

### GST Pull-Down Assay

GST and GST-fused proteins (GST-CRK-CTD, GST-RhoGDI2-WT, GST-RhoGDI2-Y24E, GST-RhoGDI2 Y130E, GST-RhoGDI2 Y153E, GST-RhoGDI2 Y24E Y130E, GST-RhoGDI2 Y24E Y153E, GST-RhoGDI2 Y130E Y153E, GST-RhoGDI2 Y24E Y130E Y153E, GST-RhoGDI2 Y24F, GST-RhoGDI2 Y130F, and GST-RhoGDI2 Y153F) were expressed in *Escherichia coli* strain BL21. The induction was performed by adding 1 mM isopropyl-β-d-thiogalactopyranoside (IPTG) to the culture with OD 1.0 at 37°C for 3 h. Whole bacteria lysates were applied to GST-BindTM Resin beads (D00172269, EMD Millipore Corp., MA, United States), and GST-tagged proteins were purified according to the manufacturer’s instructions. For pull-down experiments, JURKAT and CCRF-CEM cells were starved in medium containing 0.5% FBS. Then, 4 × 10^7^ cells were lysed in pull-down lysis buffer (25 mM Tris, pH 7.5, 150 mM NaCl, 1% Non-idet P-40, 5% glycerol, 60 mM MgCl_2_) with freshly added PMSF, and 30 μl of each sample were taken for basal control. The left lysates were incubated with 25 μg GST-tagged proteins bound to glutathione Sepharose 4B beads at 4°C for 4 h. After three washes with lysis buffer, the bound proteins were analyzed by Western blot assay.

### *In vitro* Kinase Assay

*Escherichia coli* BL21 expressing GST-RhoGDI2-WT, GST-RhoGDI2-Y24F, GST-RhoGDI2-Y130F, or GST-RhoGDI2-Y153F were induced by 1 mM IPTG, sonicated, and purified by using GST-BindTM resin beads according to the manufacturer’s instructions.

Next, 600 ng recombinant human active Src was co-incubated with 100 μM ATP, 1.2 μg of GST-RhoGDI2 (WT or mutated) fusion protein in 40 μl of kinase buffer (5 mM β-glycerophosphate, 25 mM Tris, pH 7.5, 2 mM DTT, 1 mM Na3VO4, 10 mM MgCl_2_) at 30°C for 30 min. The reactions were terminated by boiling with 10 μl of 5× Loading buffer together at 98°C for 5 min. Then samples were resolved by SDS-PAGE. CCRF-CEM cells were stimulated at 37°C with CXCL12 (25 ng/ml) for 30 min, and active ABL1 were immunoprecipitated using ABL1 antibody as previously described (17). Immunoprecipitates were washed with kinase buffer and added to the kinase buffer containing containing 2 μg of GST-RhoGDI2 mutated fusion protein.

### Western Blot Assay

JURKAT or CCRF-CEM cells (3 × 10^6^ per sample) were stimulated as described above and lysed in lysis buffer (50 mM Tris, pH 7.5, 150 mM NaCl, 1 mM EDTA, 1 mM EGTA, 1% Non-idet P-40, 2.5 mM sodium pyrophosphate, 1 mM glycerophosphate, 1 mM Na_3_VO_4_, 1 mM NaF, and 20 μg/ml aprotin/leupeptin/PMSF). Lysates were centrifuged at 4°C, 13,000 × *g* for 30 min, and the supernatants were collected. Then, 30 μg of each sample was resolved by SDS-PAGE, and proteins were transferred to nitrocellulose membranes. The membranes were washed with TBST and blocked with 5% non-fat dry milk or 3% BSA and then incubated overnight at 4°C with each indicated primary Ab and HRP-conjugated secondary Ab. Signal was detected using ECL Plus chemiluminescent detection system (Amersham).

### Statistical Analysis

Results were tested for statistical significance using Student’s *t*-test and presented as means ± *SD*. Before Student’s *t-*test, normal distribution of the data were analyzed. For groups that were not normally distributed, Mann–Whitney tests were used. Significance was determined as ^∗^*p* < 0.05, ^∗∗^*p* < 0.01, ^∗∗∗^*p* < 0.001.

## Results

### CXCR4 Is Required for ALL Cell Migration Toward CXCL12

**FIGURE 1:**
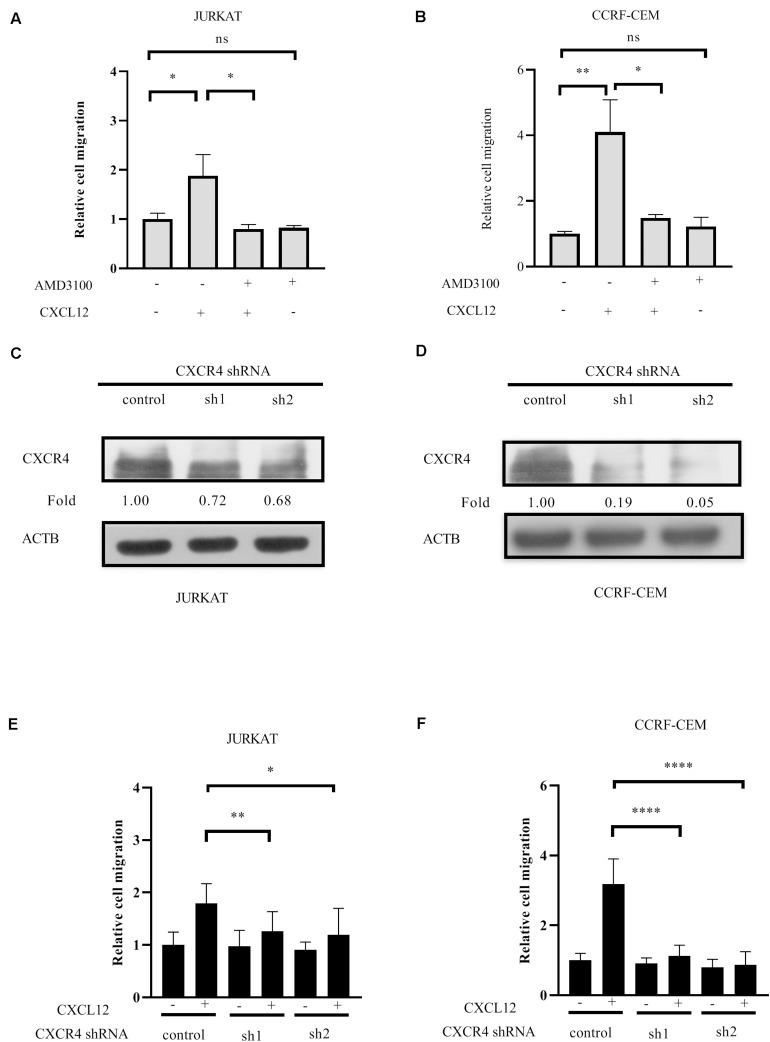
CXCR4 mediates T-ALL cell migration toward CXCL12. **(A,B)** Starved cells were pretreated with 500 ng/ml AMD3100 or equal concentration of solvent on ice for 30 min, washed with medium, and 3 × 10^5^ cells in 100 μl were used in the following transwell assay. The relative migration rate is shown. **(C–F)** Cells were infected with lentivirus containing CXCR4 shRNAs and control shRNA. Then, 72 h after infection, the interference efficiency of shRNAs to CXCR4 was detected by Western blot using CXCR4 antibody **(C,D)**, and transwell assays were conducted **(E,F)**. Each experiment was repeated at least three times. Western blot bands were analyzed using Image Pro Plus 6.0, and fold changes of CXCR4 were normalized according to ACTB. ACTB was used as the loading controls. *p < 0.05, **p < 0.01, ****p < 0.0001.

It has been demonstrated that CXCR4 is relative to infiltration of T-ALL cells (4–8, 18); meanwhile, CXCR4 cognate ligand CXCL12 is a chemokine that induces cell migration (19). To identify the role of CXCR4 in T-ALL cell migration, JURKAT and CCRF-CEM ALL cell lines were pre-incubated with or without the antagonist of CXCR4, AMD3100 in each transwell assay. The results show that AMD3100 inhibits cell migration toward CXCL12 (Figures 1A,B). After CXCR4 in JURKAT and CCRF-CEM cells was reduced by shRNAs (Figures 1C,D), another transwell assay was conducted. The results show that CXCR4 shRNAs significantly reduce cell migration toward CXCL12 (Figures 1E,F). Therefore, CXCR4 is required for T-ALL cell migration toward CXCL12.

### RhoGDI2 Inhibits CXCR4-Mediated T-ALL Cell Migration Toward CXCL12

**FIGURE 2:**
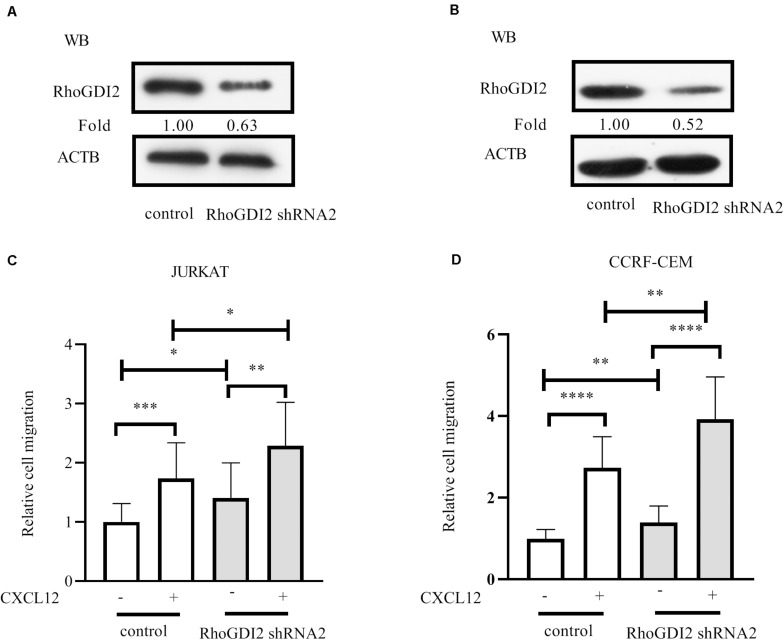
RhoGDI2 negatively regulates T-ALL cell migration toward CXCL12. **(A,B)** shRNA to RhoGDI2 or control shRNA was transfected into HEK293T to generate lentivirus, JURKAT or CCRF-CEM cells were infected, and the interference efficiency was detected by Western blot, using RhoGDI2 antibody. **(C,D)** shRNA that successfully downregulated RhoGDI2 was used in the transwell assay. Each experiment was repeated at least three times. Western blot bands were analyzed using Image Pro Plus 6.0, and fold changes of RhoGDI2 were normalized according to ACTB. ACTB was used as the loading controls. *p < 0.05, **p < 0.01, ***p < 0.001, ****p < 0.0001.

RhoGDI2 is reported to negatively or positively regulate RhoGTPases (20). We previously demonstrated that RhoA and RhoC were required for JURKAT migration toward CXCL12 (21). Therefore, we hypothesized that RhoGDI2 may play a critical role in CXCR4-mediated T-ALL cell migration. To identify the role of RhoGDI2 in the migration mediated by CXCR4, shRNAs to RhoGDI2 were generated and transfected into JURKAT or CCRF-CEM cells. Results show that RhoGDI2 sh2 significantly reduces the amount of RhoGDI2 in both cell lines (Figures 2A,B). Therefore, RhoGDI2 sh2 was used in the following transwell assay. Results show that RhoGDI2 sh2 significantly reduces the cell number migrated to CXCL12, indicating that RhoGDI2 inhibits CXCR4-mediated T-ALL migration toward CXCL12 (Figures 2C,D).

### Phosphorylation of RhoGDI2 on Y24 and Y153 Negatively Regulates Its Inhibition to CXCR4-Mediated ALL Migration by Releasing RhoA and RhoC From RhoGDI2

**FIGURE 3:**
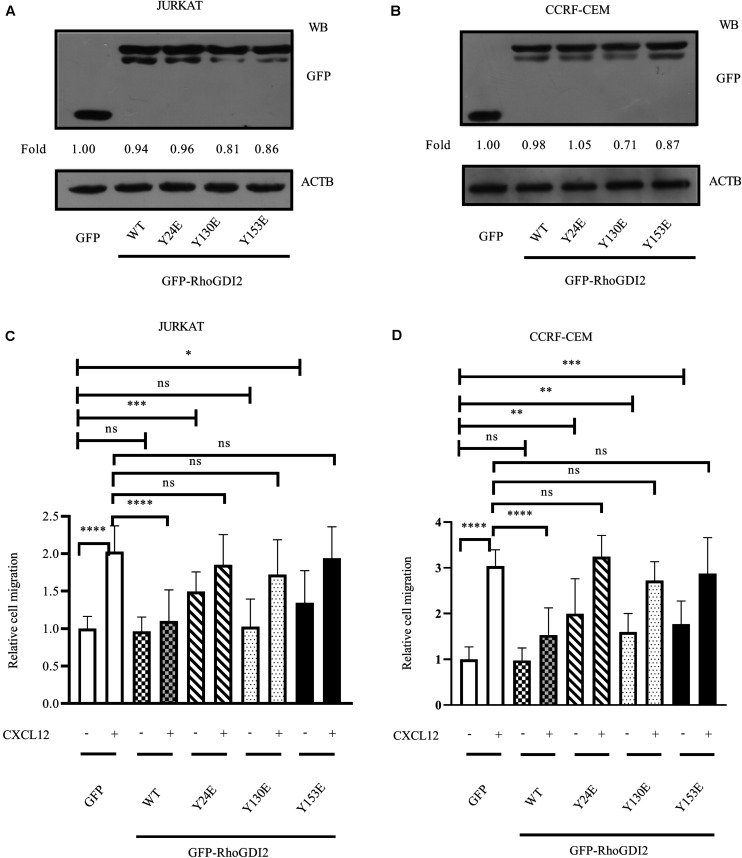
Phosphorylation of RhoGDI2 Tyr 24, 130, or 153 recovered CXCL12-mediated ALL migration. JURKAT **(A)** or CCRF-CEM **(B)** cells were infected with lentivirus containing nucleotides expressing GFP-RhoGDI2-WT, Y24E, Y130E, or Y153E, and the overexpression levels of WT or mutated RhoGDI2 were detected 48 h after infection by Western blot, using GFP antibody. **(C,D)** Transfected cells were used in the transwell assay. Each experiment was repeated at least three times. Western blot bands were analyzed using Image Pro Plus 6.0, and fold changes of GFP, GFP-RhoGDI2-WT, or mutants were normalized according to ACTB. ACTB was used as the loading controls. *p < 0.05, **p < 0.01, ***p < 0.001, ****p < 0.0001.

Previous studies report tyrosine-phosphorylation of RhoGDI2 on Y24, Y130, and Y153 (16, 22). To evaluate the effects of RhoGDI2 phosphorylation on RhoA, RhoC, and CXCL12-induced ALL migration, Y24, Y130, or Y153 were mutated into Glu (E) to mimic the phosphorylation station following relevant reference (23). Results show that phosphorylation of RhoGDI2 on Y24, Y130, or Y153 recovered CXCL12-mediated ALL migration (Figures 3A–D).

**FIGURE 4:**
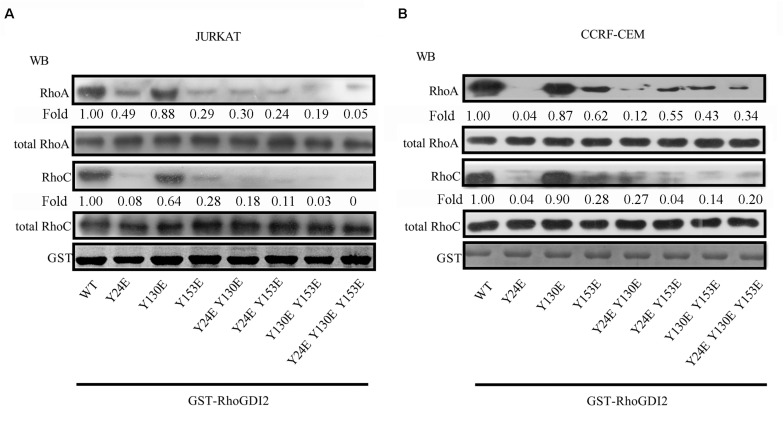
Phosphorylation of Tyr24 or Tyr153 dissociates RhoA or RhoC from RhoGDI2 and accelerates the activation of RhoA and RhoC. Cell lysates of JURKAT **(A)** or CCRF-CEM **(B)** cells were incubated with WT or mutants of GST-GDI2 in the GST pull-down assay. Then, RhoA or RhoC antibody was used in the Western blot assay to detect the level of RhoA or RhoC bound in the complex. Western blot bands were analyzed using Image Pro Plus 6.0, and fold changes of bound RhoA or RhoC were normalized according to total RhoA or RhoC. Total RhoA or RhoC was used as loading controls. GST-RhoGDI2-WT or mutants equal to that used in the GST pull-down assay were loaded and stained with coomassie blue and used as another control.

Phosphorylation of RhoGDI has been reported to stabilize the RhoA-RhoGDI complex in neutrophil cytosol (24). GST pull-down results show that RhoGDI2 24E or RhoGDI2 153E bind less RhoA and RhoC than RhoGDI2 130E does; however, they all bound less RhoA and RhoC than wild-type RhoGDI2 does (Figures 4A,B), suggesting that phosphorylation of Y24 or Y153 dissociates RhoA or RhoC from RhoGDI2 and accelerates the activation of RhoA and RhoC although the phosphorylation of Y130 has less effects on RhoA or RhoC activation.

### Src Is Activated by CXCL12 and Responsible for the Phosphorylation of RhoGDI2 on Y153

**FIGURE 5:**
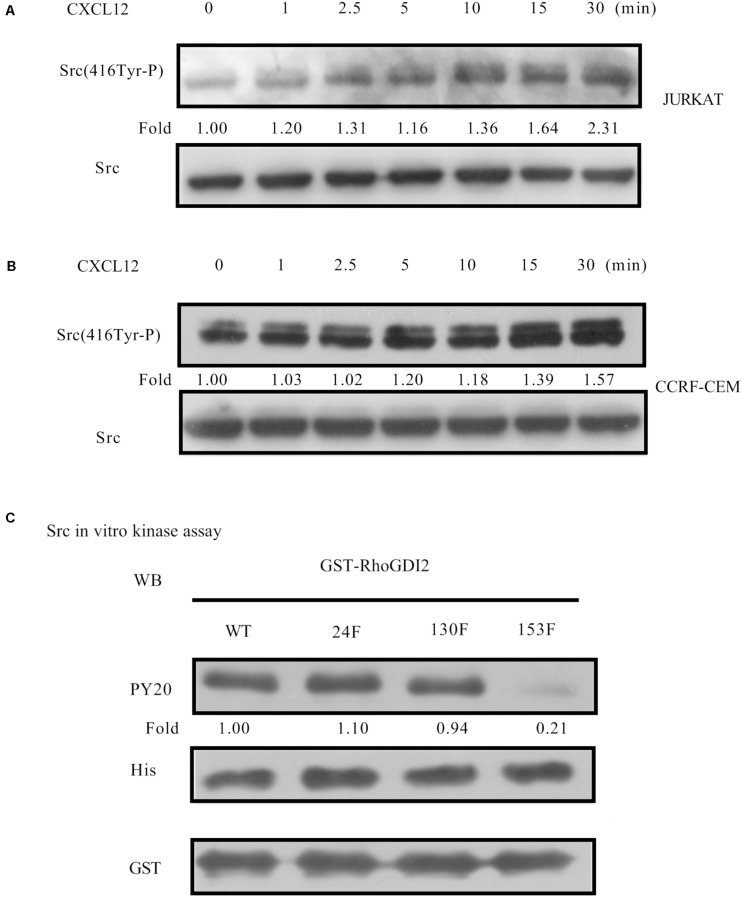
CXCL12 can activate Src in T-ALL cells. **(A,B)** Starved JURKAT or CCRF-CEM cells were incubated with CXCL12, and then cell lysates were prepared: 60 μg of cell lysates were subject to SDS-PAGE and Western blot assay, using active Src antibody, Src(416Tyr-P). **(C)** Then, 1.2 μg of GST-RhoGDI2-WT, GST-RhoGDI2-Y24F, GST-RhoGDI2 Y130F, or GST-RhoGDI2 Y153F were incubated with 600 ng recombinant human active Src (His-tag) in the *in vitro* kinase assay. Samples were boiled in 1× Loading buffer at 98°C for 5 min and then resolved by SDS-PAGE. PY20, His antibody, or GST antibody was used in the Western blot assay to detect the phosphorylation of RhoGDI2, the amount of active Src, or RhoGDI2 (WT or mutated) that were used in each *in vitro* kinase assay, respectively. Western blot bands were analyzed using Image Pro Plus 6.0. Fold changes of active Src were normalized according to total Src. Bands of phosphorylated GST-RhoGDI2 (PY20) were normalized according to WT or mutants of GST-RhoGDI2 (GST) that were used in each kinase assay.

It has been previously reported that the Src family kinase was activated and plays a role in tyrosine-phosphorylation in migration cell migration (25). CXCL12 induces migration via Src-mediated CXCR4-EGFR cross-talk in gastric cancer cells (26). Therefore, we detected the activation of Src in CXCL12-treated JURKAT and CCRF-CEM cells. The results show that Src is activated in both cell lines (Figures 5A,B), providing a prerequisite for phosphorylation of RhoGDI2. To identify which tyrosine amino acid is phosphorylated by Src, active Src and specific mutants were used in the *in vitro* kinase assay. Results show that Src is critical for the phosphorylation of RhoGDI2 on Y153 (Figure 5C).

**FIGURE 6:**
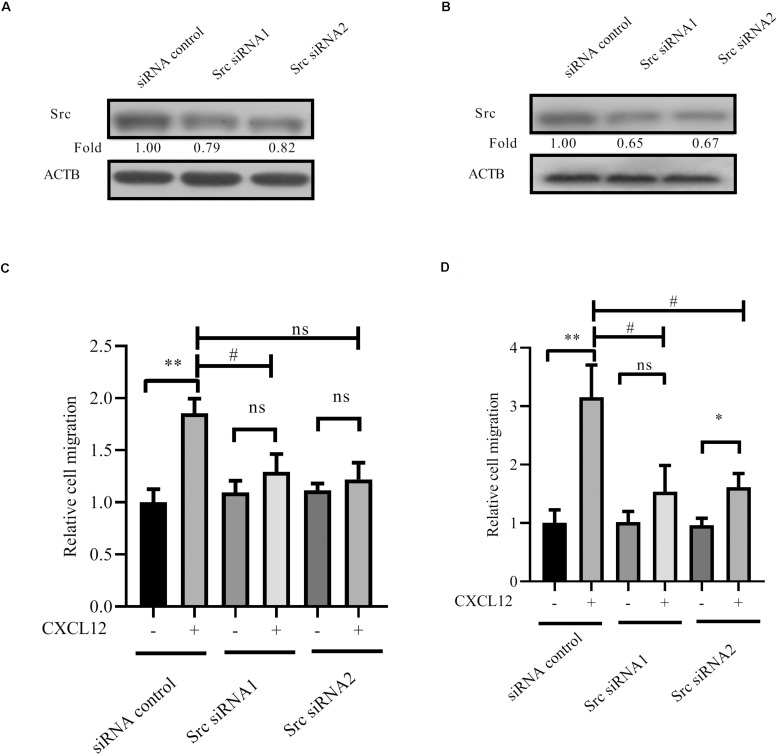
Knockdown expression of Src by siRNAs inhibited CXCR4-mediated T-ALL migration toward CXCL12. **(A,B)** siRNAs were transfected into JURKAT or CCRF-CEM cells using the methods described in the materials and methods section; 48 h after transfection, cell lysates were prepared for Western blot assay to detect the interference efficiency of siRNAs on Src. **(C,D)** Transfected cells were used in the transwell assay. Each experiment was repeated at least three times. Western blot bands were analyzed using Image Pro Plus 6.0, and fold changes of Src were normalized according to ACTB. ACTB was used as the loading controls. *p < 0.05, **p < 0.01, comparing with the negative control (CXCL12−) group. ^#^*p* < 0.05 comparing with the positive control (CXCL12+) group.

Furthermore, siRNA to Src was transfected into JURKAT and CCRF-CEM cells. Results show that migration is inhibited by siRNA (Figures 6A–D), demonstrating that Src is required for CXCR4-mediated leukemia cell migration toward CXCL12.

### ABL1 Is Activated by CXCL12 and Responsible for the Phosphorylation of RhoGDI2 on Y24 and Y130

**FIGURE 7:**
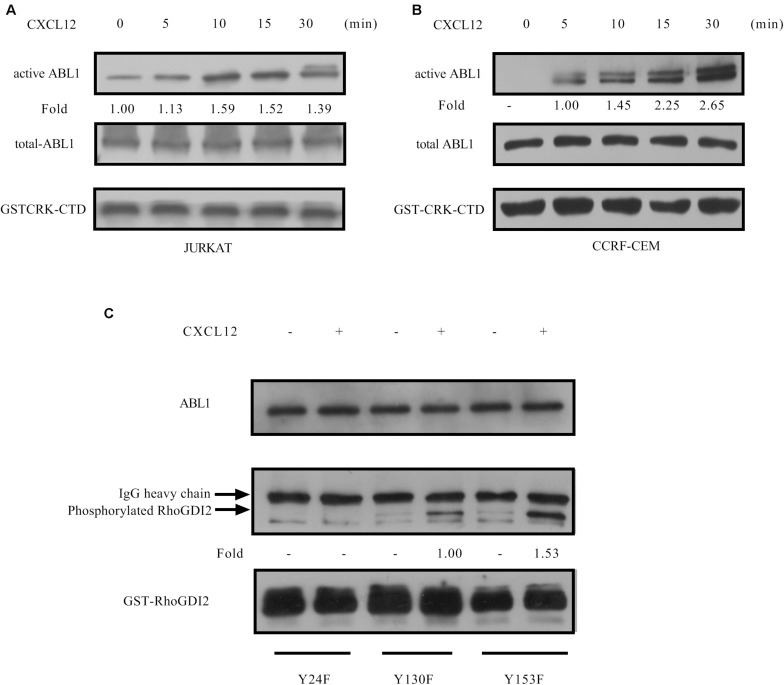
CXCL12 can activate ABL1 in T-ALL cells. **(A,B)** Starved JURKAT or CCRF-CEM cells were incubated with CXCL12, and then cell lysates were prepared: 500 μg of cell lysates were incubated with GST-Crk-CTD in the GST pull-down assay, followed by Western blot assay, using the antibody to ABL1. Western blot bands were analyzed using Image Pro Plus 6.0, and fold changes of active ABL1 were normalized according to GST-CRK-CTD. Total ABL1 was used as another loading control. **(C)** Next, 2 μg of GST-RhoGDI2-Y24F, GST-RhoGDI2 Y130F, or GST-RhoGDI2 Y153F were introduced and incubated with active ABL1 immunoprecipitated from CCRF-CEM cells treated with CXCL12 at 37°C for 30 min in the *in vitro* kinase buffer for 30 min. Samples were boiled in 1× Loading buffer at 98°C for 5 min and then resolved by SDS-PAGE. PY20, ABL1, or GST antibody was used in the Western blot assay to detect the phosphorylation of RhoGDI2, the amount of active ABL1, or RhoGDI2 (WT or mutated) that we used in each *in vitro* kinase assay, respectively. Phosphorylated RhoGDI2 were normalized according to the mutants of GST-RhoGDI2. ABL1 was used as another loading control.

GPS 5.0 predicted that Y24 may be phospholated by ABL1 (27), and KinasePhos prediction results show that Y130 may be phosphorylated by ABL1 (28). Therefore, we tested the activation of ABL1 in the CXCR4 signaling pathway and found that ABL1 is activated in response to CXCL12 (Figures 7A,B). Active ABL1 can phosphorylate RhoGDI2 on Y24 and Y130 (Figure 7C).

**FIGURE 8:**
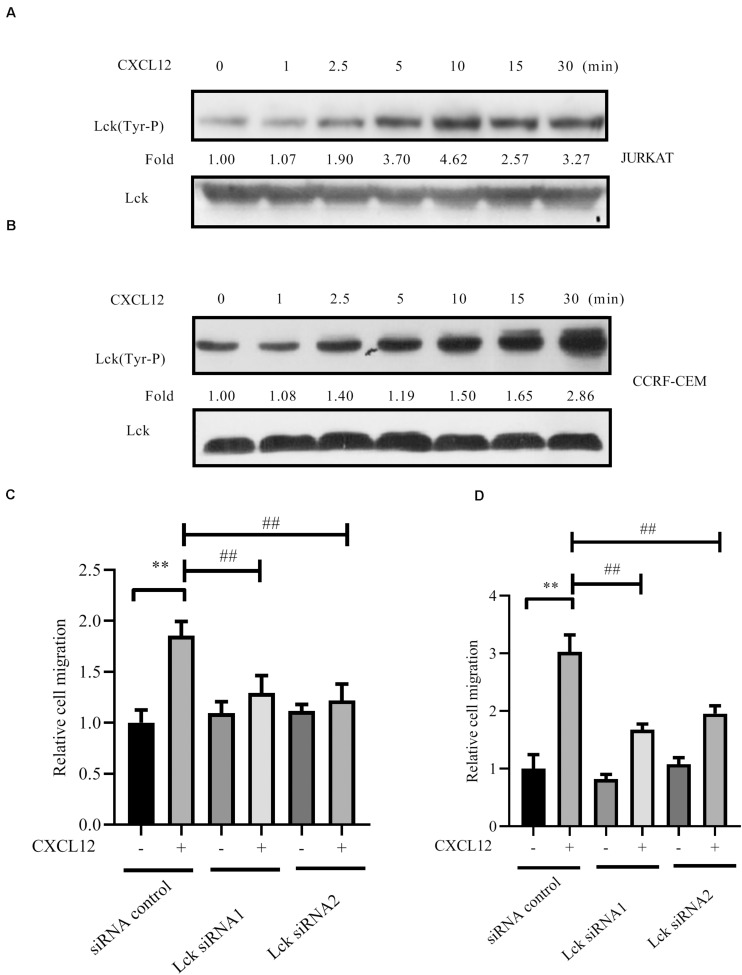
Lck was activated by CXCL12 and knocked down the expression of Lck by siRNA-inhibited CXCR4-mediated T-ALL migration toward CXCL12. **(A,B)** Starved JURKAT or CCRF-CEM cells were incubated with CXCL12, and then cell lysates were prepared: 60 μg of cell lysates were subjected to SDS-PAGE and Western blot assay, using active Lck antibody, Lck (394 Tyr-P). Western blot bands were analyzed using Image Pro Plus 6.0, and fold changes of active Lck were normalized according to total Lck. Total ABL1 was used as another loading control. **(C,D)** siRNAs were transfected into JURKAT or CCRF-CEM cells using the methods described in the materials and methods section; 48 h after transfection, cells were used in the transwell assay. Each experiment was repeated at least three times, and three repeats were included each time. **p < 0.01 comparing with the negative control (CXCL12−) group. ^##^*p* < 0.01 comparing with the positive control (CXCL12+) group.

Furthermore, Src family kinase Lck is absolutely required for the proliferation and survival of T-ALL cells that depend on NUP214-ABL1 activity (29). Therefore, activation of Lck was detected after cells were stimulated with CXCL12. Results show that Lck is activated by CXCL12 (Figures 8A,B). The migration of T-ALL cells mediated by CXCR4 is inhibited when siRNAs to Lck, whose sequences are shown in Table 1, were transfected into JURKAT or CCRF-CEM cells (Figures 8C,D).

**FIGURE 9:**
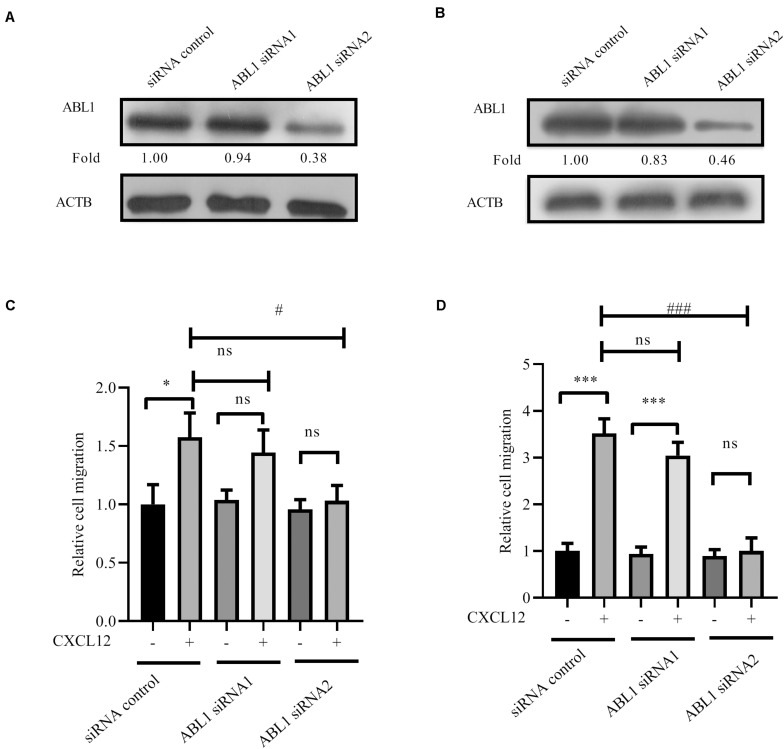
Knockdown expression of ABL1 by siRNAs inhibited CXCR4-mediated T-ALL migration toward CXCL12. **(A,B)** siRNAs were transfected into JURKAT or CCRF-CEM cells using the methods described in the materials and methods section; 48 h after transfection, cell lysates were prepared for Western blot assay to detect the interference efficiency of siRNAs on ABL1. Western blot bands were analyzed using Image Pro Plus 6.0, and fold changes of ABL1 were normalized according the loading control ACTB. **(C,D)** Transfected cells were used in the transwell assay. Each experiment was repeated at least three times. *p < 0.05, ***p < 0.001, comparing with the negative control (CXCL12−) group. ^#^*p* < 0.05, ^###^*p* < 0.001, comparing with the positive control (CXCL12+) group.

Finally, siRNA to ABL1 was transfected into JURKAT and CCRF-CEM cells. Results show that migration is inhibited by siRNA (Figures 9A–D), demonstrating that ABL1 is required for CXCR4-mediated leukemia cell migration toward CXCL12.

**FIGURE 10:**
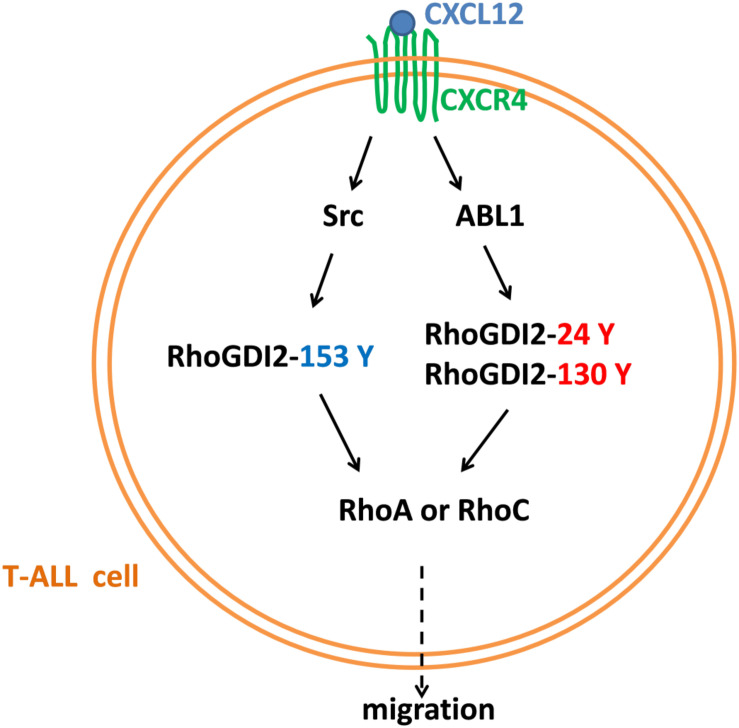
A diagram of the mechanism of CXCR4-mediated T-ALL migration.

Taken together, RhoGDI2 is a critical signal molecule downstream of CXCR4. CXCL12/CXCR4 triggers the activation of downstream kinases, including Src and ABL1 responsible for the phosphorylation of RhoGDI2 on Y153, Y24, and Y130. Phosphorylation of RhoGDI2 on Y24 and Y153 releases RhoA and RhoC from RhoGDI2, which recovers CXCR4-mediated migration toward CXCL12 although the phosphorylation of Y130 has less effect on RhoA or RhoC binding. Therefore, RhoGDI2 may be a brake for CXCR4-positive T-ALL migration (Figure 10).

## Discussion

Xenotransplantation of NSG mice with Jurkat or CCRF-CEM cells demonstrates that both can infiltrate the CNS at similar proportions and that antagonizing CXCR4 prevents the clinical features of the disease and T-ALL cell CNS tropism (4). Pediatric acute lymphoid leukemia-phase II (TARGET, 2018) data from cBioPortal for cancer genomics (30, 31) reveals that RhoGDI2 gene *ARHGDIB* deep depletion is closely relative to the bone marrow site of relapse in B-ALL. However, due to the restriction of the database source, the role of RhoGDI2 in T-ALL progression remains to be investigated. Here, we find that RhoGDI2 is phosphorylated by Src and ABL1 in response to CXCR4 stimulation by CXCL12 in T-ALL cell lines and that RhoGDI2 decreases the CXCR4-mediated T-ALL cell line migration toward CXCL12 (Figures 1, 2). Because migration is necessary for infiltration and consequent relapse of leukemia, it would be interesting to explore whether RhoGDI2 brakes CXCR4-mediated T-ALL infiltration *in vivo*.

Recent reports show that RhoGDI2 participates in β1 inside-out signaling (16) and β2 outside-in signaling (20) in T lymphocytes. Phosphorylations of p85-bound RhoGDI2 on Y130 and Y153 by c-Abl and Src are required for the complex to be recruited to PSGL1 and thereby regulates β1 integrin-mediated T cell adhesion to VCAM-1 (16). Upon P-selectin glycoprotein ligand-1 engagement, RhoGDI2 is phosphorylated at Y24 and Y153 by Src, ABL1, and Syk (20). In bladder cancer, it was demonstrated that, except Y153, Y24 was also the primary Src phosphorylation site (22). The Y24 mutant also showed a partial reduction in phosphorylation by Src, which was significant in HEK293T but noticeably less in UMUC3 human bladder cancer cells. Src phosphorylation in RhoGDI2 has been involved in migration of neutrophils toward fMLP (24). In our *in vitro* kinase assay, results show that active Src primarily activates Y153 rather than Y24 of RhoGDI2 (Figure 5C).

Proteomic analyses have identified phosphorylation in Y24 of RhoGDI2 in pervanadate-treated JURAT cells, an established leukemic T-cell line (32, 33). Using JURKAT and CCRF-CEM T-ALL cell lines, we find that RhoGDI2 Y24 and Y130 are phosphorylated by ABL1 (Figure 7C). Although ABL1 is reported to phosphorylate Y130 of p85-bound RhoGDI2 upon PSGL-1 ligation (16), we haven’t found any scholarly report that ABL1 phosphorylation in Y24 and Y130 of RhoGDI2 is involved in the migration of T-ALL cells toward CXCL12, suggesting the involvement of ABL1 in T-ALL. Consistently, the NUP214-ABL1 fusion protein, which is a constitutively active form of ABL1, is found in 6% of patients with T-ALL and promotes proliferation and survival of T-lymphoblasts (34). Here, we found that Src and ABL1 are involved in the migration of the T-ALL cell lines that is mediated by CXCR4 (Figures 6, 9). These findings underscore the potential of Src kinase inhibitors and of the dual ABL1/Src kinase inhibitors dasatinib and bosutinib for the treatment of T-ALL. Additionally, knockdown of PRL-3, highly expressed in T-ALL patients, significantly impedes T-ALL cell migration capacity *in vitro* and *in vivo* (35). The Src signaling pathway is affected by PRL-3 although Src is not a direct substrate of PRL-3 (35). Coincidentally, in a mass spectrometry experiment to identify protein interaction partners of ABL1, we found that RPL-3 was in the immunoprecipitation complex of ABL1 (unpublished data). We speculate that ABL1 may be a cooperator of RPL-3 to regulate Src. However, this hypothesis needs further demonstration.

RhoGDI1 Tyr156 is the major site of Src phosphorylation, whose phosphorylation decreases its affinity for RhoGTPase, including Rac1, Cdc42, and RhoA (23). Phosphorylation on Tyr156 in RhoGDI abrogates the ability of RhoGDI1 to rebind and inhibit activated Rho GTPase both *in vitro* and in intact cells (23). Tyrosine-phosphorylated RhoGDI1 localizes to areas of membrane ruffling and to podosome rosettes in Src-transformed NIH-3T3 fibroblasts (23). Phosphorylation of Rho GDI1 stabilizes the RhoA-RhoGDI1 complex in neutrophil cytosol (24). RhoGDI2 contains an equivalent tyrosine residue at aa 153. Phosphorylation decreases the amount of Rac1 in RhoGDI2 complexes and increases RhoGDI2 association with cell membranes (22). Although it has been reported that RhoGDI2 positively regulates the activation of Rac1 and Cdc42 (20), we find the phosphorylation on 24 and 153 tyrosine complex less RhoA or RhoC in human T-ALL cell lines (Figure 3), raising a possibility to release the negative regulatory role of RhoGDI2 in T-ALL cell migration toward CXCL12 (Figure 4).

## Data Availability Statement

The raw data supporting the conclusions of this article will be made available by the authors, without undue reservation.

## Author Contributions

JL and JW designed the study, collated the data, and carried out data analyses. JW and HZ conducted the experiments. JL produced the initial draft of the manuscript. JW and LW contributed to drafting the manuscript. All authors have read and approved the final submitted manuscript.

## Conflict of Interest

The authors declare that the research was conducted in the absence of any commercial or financial relationships that could be construed as a potential conflict of interest.
